# Gut microbiota microbial metabolites in diabetic nephropathy patients: far to go

**DOI:** 10.3389/fcimb.2024.1359432

**Published:** 2024-05-08

**Authors:** Jian-Xiu Yu, Xin Chen, Su-Gang Zang, Xi Chen, Yan-Yan Wu, Li-Pei Wu, Shi-Hai Xuan

**Affiliations:** Medical Laboratory Department, Affiliated Dongtai Hospital of Nantong University, Dongtai, Jiangsu, China

**Keywords:** diabetic nephropathy, gut microbiota, microbial metabolites, diagnosis and treatment, therapeutic strategies

## Abstract

Diabetic nephropathy (DN) is one of the main complications of diabetes and a major cause of end-stage renal disease, which has a severe impact on the quality of life of patients. Strict control of blood sugar and blood pressure, including the use of renin–angiotensin–aldosterone system inhibitors, can delay the progression of diabetic nephropathy but cannot prevent it from eventually developing into end-stage renal disease. In recent years, many studies have shown a close relationship between gut microbiota imbalance and the occurrence and development of DN. This review discusses the latest research findings on the correlation between gut microbiota and microbial metabolites in DN, including the manifestations of the gut microbiota and microbial metabolites in DN patients, the application of the gut microbiota and microbial metabolites in the diagnosis of DN, their role in disease progression, and so on, to elucidate the role of the gut microbiota and microbial metabolites in the occurrence and prevention of DN and provide a theoretical basis and methods for clinical diagnosis and treatment.

## 1 Introduction

Diabetes is one of the most common chronic diseases worldwide, with prevalence and incidence rates increasing annually (Liu et al., 2021). It is estimated that by 2045, the absolute number of diabetes patients will increase by 46% (Sun H. et al., 2022). Diabetes can cause various serious and some life-threatening complications (Popoviciu et al., 2023). Diabetic nephropathy (DN) is one of the common microvascular complications, characterized by structural and functional damage to the kidneys (Wu and Huang, 2023). Clinical manifestations include massive proteinuria, hypertension, and edema, and it is one of the main causes of end-stage renal disease (ESRD) (Wu et al., 2023). At present, the diagnosis of DN depends on a decreased glomerular filtration rate (GFR) or increased urinary albumin excretion (UAE), but these changes are not unique to DN, and the diagnostic sensitivity and specificity in the preclinical stage of diabetic kidney damage are also limited (Oshima et al., 2021). At present, the treatment of DN mainly involves lifestyle guidance, metabolic therapy, and hypoglycemic and antihypertensive drugs to help patients slow down disease progression, thereby improving their quality of life (Liu P. et al., 2023). However, due to the complex pathogenesis of DN, no breakthrough progress has been made in the treatment of DN. Therefore, there is an urgency to search for new biomarkers generated by the pathogenesis of this disease to assist in its diagnosis, follow-up, treatment, and prognosis.

The human intestine harbors a variety of microorganisms, such as bacteria, fungi, and viruses, that are involved in the digestion of food, synthesis of essential vitamins and amino acids, elimination of pathogens, and clearance of toxins (Fernandes et al., 2022; Xiang et al., 2023). Through metagenomic sequencing analysis of human fecal samples, intestinal flora such as Bacteroidetes, Firmicutes, Proteobacteria, Actinobacteria, Verrucomicrobia, Cyanobacteria, and Spirochaetes have been identified (de Vos et al., 2022; Chen et al., 2023). Many studies have shown that changes in the abundance, diversity, and colonization location of the gut microbiota and alterations in serum metabolites can lead to DN, diabetic retinopathy, diabetic cardiovascular disease, and other complications (Lv et al., 2022; Xu et al., 2022). However, the specific role of the gut microbiota in DN is not yet fully understood. The recent emergence of the gut–kidney axis theory has gradually revealed the correlation between gut microbiota and kidney diseases (Paul et al., 2022). The gut microbiota of DN patients is significantly different from that of healthy individuals, with a decrease in beneficial bacteria, such as *Bifidobacterium* and *Lactobacillus*, and an increase in the number of pathogenic bacteria, such as *Enterobacter* (Hu et al., 2020; Castillo-Rodriguez et al., 2018). An imbalance of the gut microbiota can lead to intestinal barrier damage, increased intestinal permeability, and accelerated transfer of microbial metabolites (such as indoxyl sulfate and p-cresyl sulfate) into the bloodstream, exacerbating kidney damage (Chen et al., 2023). Imbalance of the gut microbiota leads to metabolic endotoxemia, which induces chronic inflammation, short-chain fatty acid (SCFA) metabolism, oxidative stress, and other factors that affect the development of DN (Li J. et al., 2022). Correcting the imbalance of the gut microbiota may be a new target for treating DN.

We summarize the characteristics of the gut microbiota and metabolism in DN patients and discuss the application of gut microbiota and metabolism as biomarkers in DN, the role of the gut microbiota and metabolism in disease occurrence and development, and the application of microbial targeted therapy in DN.

## 2 Gut microbiota in DN patients

### 2.1 Alteration of gut microbiota in DN Patients

The stability of the gut microbiota is closely related to host health and disease (Gebrayel et al., 2022). Normal gut microbiota contains a large number of bacteria such as *Bacteroides*, *Bifidobacterium*, and *Lactobacillus*. DN patients have imbalances in gut microbial composition, abundance, and diversity (Kikuchi et al., 2019). In 20 patients with type 2 diabetes (T2DM) and chronic kidney disease (CKD), the gut microbiota showed significantly higher levels of Proteobacteria, Verrucomicrobia, and Fusobacteria, which can produce lipopolysaccharides (LPS), compared with a health control group (Salguero et al., 2019). Tao et al. (2019) also found a high abundance of Proteobacteria in 14 confirmed cases of DN. Cai et al. (2022) also found high abundance of Proteobacteria in nondialysis-dependent DN patients. In addition, compared with healthy controls, the relative abundance of Ruminococcaceae, *Butyricicoccus*, and Lachnospiraceae, which produce SCFAs, was reduced in 31 nondialysis-dependent patients. Shang et al. (2022) found that the gut microbiota of 180 DN patients was enriched in Proteobacteria, Actinobacteriota, Synergistota, Euryarchaeota, Patescibacteria, Verrucomicrobiota, and Cyanobacteria, compared with healthy controls, while Bacteroidota and Bacteria unclassified were depleted. Compared with healthy controls, there was a decrease in the abundance of Firmicutes in 20 patients with DN, while Corynebacteriales and *Eisenbergiella*, as well as *Ralstonia*, were enriched (Song et al., 2021). In a study involving 60 patients with DN, there was no significant difference in the relative abundance of Actinobacteria and Firmicutes between the DN and healthy control group (Chen et al., 2021). That study confirmed that *Alistipes*, *Bacteroides*, *Subdoligranulum*, *Lachnoclostridium*, and *Ruminococcus torques* were detrimental factors in the development of DN (Chen et al., 2021). Compared with healthy controls, the gut microbiota of 43 patients diagnosed with stage 3 or 4 DN was enriched in *Haemophilus*, *Escherichia–Shigella*, *Megalococcus*, *Veillonella*, and *Anaerostipes* (Du et al., 2021). Butyrate-producing bacteria (*Clostridium*, *Ruminococcus*, and *Eubacterium*) and potential probiotics (*Lactobacillus* and *Bifidobacterium*) were significantly reduced in T2DM and DN patients (Zhang L. et al., 2022). Compared with T2DM patients without kidney damage for >10 years, 35 confirmed cases of DN showed a significant increase in the abundance of *Christensenella*, *Clostridium-XIVa*, *Eisenbergiella*, *Flavonifractor*, and *Clostridium-XVIII*, while the abundance of butyrate-producing bacteria, *Bacillus*, *Enterobacter*, *Trichospira*, and *Roseburia* was significantly reduced (Lu et al., 2023). Whole-genome analysis showed enrichment of seven bacterial species in the feces of 15 DN patients, including *Alistipes shahii*, *Alistipes communis*, *Alistipes onderdonkii*, *Bacteroides intestinalis*, *Ruminococcus* sp. *strain JE7A12*, and *Odoribacter splanchnicus* (Kim et al., 2023). However, whole-genome analysis of European women showed that *A. shahii* was higher in the healthy control group than in the diabetes group (Dwiyanto et al., 2021), which may be due to racial, dietary, and geographical differences (Gaulke and Sharpton, 2018). Differences in lifestyle, diet, race, and medical conditions may be the main factors leading to differences in gut microbiota expression in DN (Dwiyanto et al., 2021). Therefore, long-term, multicenter research is still needed to help us better understand the relationship between the gut microbiota and DN (**Table 1**).

**Table 1:** Alteration of gut microbiota in DN.

Studies	Subjects	The variety of Gut microbiota	Research method
Song et al. (2021)	DN patients	**Increased:** **At the genus level:** *Eisenbergiella*, *Ralstonia*, *Intestinimonas*, *Eubacterium_fissicatena_group* **Decreased:** **At the phylum levels:** Firmicutes	High-throughput sequencing
Chen et al. (2021)	DN patients	**Increased:** **At the genus level:** *Alistipes*, *Bacteroides*, *Subdoligranulum*, *Lachnoclostridium*, *Parabacteroides* **Decreased:** *Klebsiella*	High-throughput sequencing
Salguero et al. (2019)	DN patients	I**ncreased:** **At the phylum levels:** Proteobacteria, Verrucomicrobi, Fusobacteria **Decreased:** **At the phylum levels:** Firmicutes	16sRNA
Tao et al. (2019)	DN patients	**Increased:** **At the phylum levels:** Proteobacteria **At the genus level:** *Coriobacteriaceae*, *Escherichia-Shigella* **Decreased:** **At the genus level:** *Prevotella_9*	16sRNA
Du et al. (2021)	DN patients	**Increased:** **At the phylum levels:** Actinobacteria **At the class levels:** Actinobacteria, Bacilli, Coriobacteriia, Negativicutes **At the order levels:** Betaproteobacteriales, Bifidobacteriales, Coriobacteriales, Lactobacillales, Selenomonadales **At the family level:**Atopobiaceae, Bifidobacteriaceae, Burkholderiaceae, Lactobacillaceae, Streptococcaceae, Tannerellaceae, Veillonellaceae **At the genus level:** *Acidaminococcus*,Lactobacillus, *Megasphaera, Mitsuokella, Olsenella, Prevotella_7, Sutterella* **Decreased**: **At the class levels:** Alphaproteobacteria, Clostridia **At the order levels:** Chitinophagales, Clostridiales, Rhizobiales, Xanthomonadales **At the family level:**Chitinophagaceae, Lachnospiraceae, Rhodanobacteraceae **At the genus level:** *Lachnoclostridium, Roseburia, Tyzzerella_3*	16S rDNA
Zhang L. et al. (2022)	DN patients	**Increased:** **At the genus level:** *Bacteroides, Bacteroides stercoris, Prevotella* sp. *MSX73, Barnesiella, Alistipes ihumii, Bacteroides stercoris CAG_120, Tannerella* sp. *CAG_51, Parabacteroides* sp. *20_3* **At the species level**:*Bacteroides stercoris, Bacteroides_eggerthii* **Decreased:** **At the genus level:** *Prevotella, Lachnospira, oseburia intestinalis, Bacteroides plebeius CAG_211, Clostridium* sp. *CAG_768, Fusobacterium varium, Clostridium* sp. *26_22, Eubacterium* sp. *AF22_9, Roseburia* sp. *AM23_20* **At the species level**:*Bacteroides fragilis*	Metagenomic sequencing
Lu et al. (2023)	DN patients	**Increased:** **At the genus level**:*Christensenella*, *Clostridium-XIVa*, *Eisenbergiella*, *Flavonifractor*, *Clostridium-XVIII* **Decreased:** **At the genus leve**l:*butyric-producing bacteria*, *Bacillus*, *Enterobacter*, *Trichospir*a, *Rosacella*	16S rDNA
Kim et al. (2023)	DN patients	**Increased:** **At the species level**:*Alistipes onderdonkii*, *Alistipes shahii*, *Alistipes communis*, *Ruminococcus* sp. strain JE7A12, *Bacteroides intestinalis*, and *Odoribacter splanchnicus*	Metagenomic sequencing
Cai et al. (2022)	DN patients	**Increased:** **At the phylum levels:** Proteobacteria **At the class levels:** δ-proteobacteria, γ-probacteria, **At the order levels:** Pseudomonadales, Desulfovibrionales **At the family levels:** Moraxellaceae, Desulfovibrionaceae **At the genus levels:** *Acinetobacter*, *Desulfovibrio*, *Erysipelatoclostridium*, *Hungatella*, **Decreased:** **At the phylum levels:** Firmicutes **At the class levels:** Clostridia **At the order levels:** Clostridiales **At the family levels:** Ruminococcaceae, Lachnospiraceae **At the genus levels:** *Ruminococcaceae_UCG_013*, *Lachnospira*, *Ruminococcaceae_UCG_014*, *Ruminococcaceae_UCG_003*, *Butyricicoccus*, *Lachnospiraceae_NK4A136_group*, *Eubacterium*	16S rDNA
Zhang B. et al. (2022)	DN rats	**Increased:** **At the phylum levels:** Actinobacteriota **At the class levels:** Bacilli, Bacteroidia **At the order levels:** Lactobacillales, Erysipelotrichales **At the family levels:** *Lactobacillaceae* **At the genus levels:** *NK4A214_group* **Decreased:** **At the phylum levels:** Firmicutes **At the class levels:** Clostridia **At the order levels:** Clostridiales, Clostridia UCG-014 **At the genus levels:** *Lachnospiraceae_NK4A136_group*, *Romboutsia*	16S rRNA
Wu et al. (2022)	DN rats	**Increased:** **At the genus levels:** *Negativibacillus*, *Rikenella* **Decreased:** **At the genus levels:** *Akkermansia*, *Candidatus*, *Erysipelatoclostridium*, *Ileibacterium*	16s rDNA

### 2.2 The diagnostic and early warning value of microbiota in DN patients

The gut microbiota composition in DN patients undergoes significant changes, which can serve as biomarkers to differentiate clinical diagnosis or confirm DN through biopsy. For patients who are contraindicated for renal biopsy, gut microbiota testing may be a crucial alternative solution (Shang et al., 2020). Among the 14 DN patients confirmed by biopsy in Sichuan, China, the genus *Prevotella_9* accurately distinguished DM patients from healthy controls, with an area under the receiver operating characteristic curve (AUC) of 0.900. *Escherichia–Shigella* and *Prevotella_9* also accurately differentiated DN patients confirmed by biopsy from DM patients, with an AUC of 0.860, which aided in the diagnosis of DN (Tao et al., 2019). However, Lu et al. found different results in 35 cases of DN confirmed by biopsy in Shanxi, China, where *Flavonifractor* (AUC=0.909) or *Eisenbergiella* (AUC=0.886) accurately identified DN and DM patients (Lu et al., 2023), which may be related to differences in northern and southern regions and dietary habits. *Clostridium* sp. CAG_768 (AUC=0.941), *Bacteroides propionicifaciens* (AUC=0.905), and *Clostridium* sp. CAG_715 (AUC=0.908) effectively differentiated DN patients from the healthy control group. Multiple linear regression analysis showed that the combined detection of *Fusobacterium varium*, Pseudomonadales, and *Prevotella* sp. MSX73 (AUC=0.889) distinguished T2DM from DN, and the AUC of bacterial biomarkers for T2DM and DN was higher than urinary albumin to creatinine ratio (ACR), albumin, and urinary creatinine ratio (Zhang L. et al., 2022). A random forest model constructed from the 25 least correlated microbial genera had an AUC of 0.972, indicating a high predictive ability of gut microbiota for DN (Du et al., 2021). These results suggest that the gut microbiota may be promising candidates for diagnosing DN. However, current research shows that the biomarkers of gut microbiota used for diagnosing DN vary among regions and races (Gaulke and Sharpton, 2018). Therefore, more clinical research is needed to explore the value of gut microbiota in DN diseases.

### 2.3 Gut microbiota associated with occurrence and development of DN

**Figure 1:**
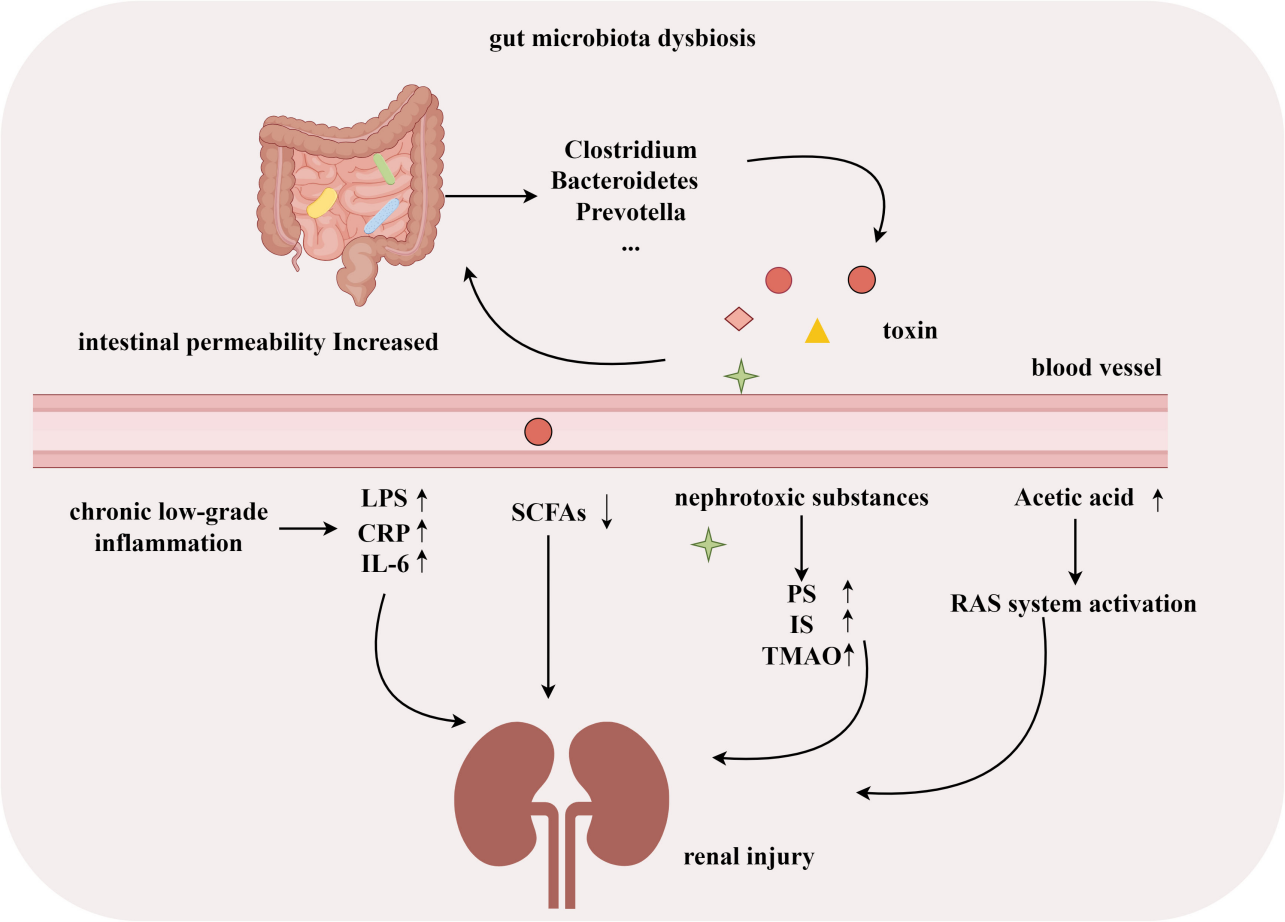
Gut microbiota associated with development of DN. (By Figdraw).

Many studies have shown significant changes in the gut microbiota of patients with DN. Dysbiosis of the gut microbiota in DN patients is associated with endotoxemia, inflammation (Zhang et al., 2021), intestinal barrier dysfunction (Xiong et al., 2019; Sun X. et al., 2022; Xu et al., 2022), and a decrease in beneficial bacteria that produce SCFAs (Sabatino et al., 2017). Pathogenic bacteria, such as *Clostridium*, *Bacteroides*, and *Prevotella*, can increase intestinal barrier permeability by producing toxins (Das et al., 2021). Increased intestinal permeability promotes the reabsorption of ammonia, and toxins produced by microbial metabolism (such as indoxyl sulfate and p-cresyl sulfate) are transferred into the blood, exacerbating kidney damage (Lv et al., 2022). Microbial dysbiosis, mainly characterized by an overgrowth of *Proteus*, is associated with increased inflammation in DN patients and a decrease in SCFA-producing bacteria, which is a key factor in the pathogenesis of DN (Salguero et al., 2019; Stavropoulou et al., 2021). In a DM rat model, excess acetate produced by dysbiosis of the gut microbiota induced early kidney damage by activating the renal renin–angiotensin system (Lu et al., 2020). In experimental models of diabetes, microbiota-derived phenyl sulfate (PS) is associated with the progression of albuminuria (Kikuchi et al., 2019). Several recent studies have shown that regulating gut microbiota dysbiosis and improving intestinal barrier function can effectively reduce uremic toxin levels and serum proinflammatory mediators [such as tumor necrosis factor-α, interleukin (IL)-1β, and IL-18], thereby delaying the progression of DN (Han et al., 2023; Shi et al., 2023; Wang et al., 2023; Wu et al., 2023). These studies indicate that gut microbiota disorders play an essential role in the development of DN, and further exploration is needed to diagnose or treat DN by targeting the composition of gut microbiota (**Figure 1**).

## 3 Microbial metabolites in DN patients

### 3.1 Alteration of metabolites in DN patients

The interaction between gut microbiota and the host is mainly achieved through the production of metabolites, which play a key role in the pathogenesis of DN by producing a large number of metabolites (Zhang B. et al., 2022). Zhu et al. (2022) have shown that amino acid metabolism may play an important role in the progression of DM and DN. N-Acetylaspartic acid, L-valine, betaine, isoleucine, asparagine, and L-methionine are upregulated in patients with T2DM and DN, with a more significant increase in the latter. High levels of L-leucine and isoleucine are significantly correlated with rapid estimated GFR decline. Compared with healthy controls, DN patients have elevated levels of stearic acid, glutaric acid, 2-Amino-3-methylimidazo(4,5-f) quinoline, and L-proline, and decreased levels of 1,3,7-trimethyluric acid, homocarnosine, epinephrine, N-acetylputrescine, linoleic acid, and ephedrine (Chen et al., 2023). In addition, the abundance of SCFA metabolites, valerate, and caproate, are significantly decreased in the serum of DN patients (Zhong et al., 2022). Compared with healthy controls, 11 DN patients had significantly higher levels of leucine, isoleucine, methionine, valeric acid, and phenylacetate, and lower levels of acetate (Kim et al., 2023). Li Y. et al. (2022) have also found decreased levels of acetate in DN patients. Acetate is one of the main components of SCFAs, and the levels of other SCFAs components, propionate, and butyrate, are lower in DN patients compared with DM patients and healthy controls. This may be related to the decrease in SCFA-producing bacteria such as Ruminococcaceae, Lachnospiraceae, and Bacteroidaceae in the gut microbiota of DN patients (Chen T. et al., 2022). However, the construction of DN rat models showed that serum acetate levels increase in DM rats, accompanied by increased proteinuria, and *in vitro* experiments have confirmed that excessive acetate can cause tubulointerstitial damage (Hu X. et al., 2020; Lu et al., 2020). This difference may be related to different research subjects and diseases, and multicenter and cross-racial studies are needed to confirm the role of SCFAs in DN. Gut microbiota metabolites, such as PS and trimethylamine-N-oxide, are typical uremic toxins associated with podocyte injury (Fernandes et al., 2019) (**Table 2**).

**Table 2:** Alteration of metabolic Changes in DN.

Studies	Subjects	The variety of Gut microbiota	Research method
Zhong et al. (2022)	DN patients	**Decreased:** valerate, caproate	GC–MS
Balint et al. (2023)	DN patients	**Increased:** indoxyl sulfate, Butenoylcarnitine, Sorbitol, Dimethyl Arginine **Decreased:** arginine, hippuric acid	UHPLC-QTOF-ESI-MS Analysis
Chen et al. (2023)	DN patients	**Increased:** Stearic acid, Glutaric acid, 2-Amino-3-methylimidazo[4,5-f]quinoline, L-Proline **Decreased:** 1,3,7-Trimethyluric acid, Homocarnosine, Epinephrine, N-Acetylputrescine, Linoleic acid, Ephedrine	UPLC-MS/MS
Peng et al. (2022)	DN patients	**Increased:** L-homocys, 3-sulfinylpyruvate, 2,3-Diketo-5-methythiopentyl-1-phosphate, dehydroalanine, L-cysteine, s-adenosyl-L-methionine, s-methyl-5-thio-D-ribose 1-phosphate, sn-Met-Cys-Ser, Asn-Cys-Pro-Pro **Decreased:** Mercaptopyruvate,	untargeted LC/MS
Kim et al. (2023)	DN patients	**Increased:** valine, isoleucine, methionine, valerate, phenylacetate **Decreased:** acetate	NMR spectroscopy
Shi et al. (2023)	DN patients	**Increased:** urinary metabolites propionic acid, oxoadipic acid, leucine, isovaleric acid, isobutyric acid, and indole-3-carboxylic acid	UPLC-MS/MS
Zhang B. et al. (2022)	DN rats	**Increased:** isomaltose, D-mannose, galactonic acid, citramalic acid, prostaglandin B2 **Decreased:** 3-(2-Hydroxyethyl) indole, 3-methylindole, indoleacrylic acid	UHPLC-QE-MS
Trifonova et al. (2022)	DN patients	**Increased:** L-arginine, L-proline, L-cysteine, citrulline, 4-guanidinobutanamide, N2-succinyl-L-ornithine, creatinine, citrulline, phosphoglycolic, 2-oxo-3-hydroxy-4-phosphobutanoic acids **Decreased:** creatine, thiosulfate, thiocysteine, 3-sulfinylpyruvic acid	MS/MS
Zhu et al. (2022)	DN patients	**Increased:** N-acetylaspartic acid, L-valine, isoleucine, asparagine, betaine, L-methionine	LC–MS
Winther et al. (2020)	DN patients	**Increased:** indoxyl sulphate, L-citrulline. Homocitrulline, L-kynurenine **Decreased:** tryptophan	HPLC MS/MS
Wu et al. (2022)	DN rats	**Increased:** D-arabinose 5-phosphate, estrone 3-sulfate, L-theanine, 3′-aenylic acid, adenosine 5′-monophosphat **Decreased:** aurohyocholic acid sodium salt, calcium phosphorylcholine chloride, tauro-alpha-muricholic, sodium salt, galactinol, phosphocholine	LC-MS

### 3.2 The diagnostic and early warning value of metabolites in DN patients

Enrichment analysis has confirmed the involvement of the urea cycle, TCA cycle, glycolysis, and amino acid metabolism in the pathogenesis of DN. Meta-analysis of existing studies on DN identified lactate, hippuric acid, urea (in urine), and glutamine (in blood) as the most important noninvasive early diagnostic biomarkers (Roointan et al., 2021). Random forest model analysis showed that methionine and branched-chain amino acids (AUC=0.832) were among the most significant features, second only to estimated GFR and proteinuria, for distinguishing between DN patients and healthy controls (Kim et al., 2023). Zhu et al. (2022) confirmed that high levels of L-leucine (AUC=0.834) and isoleucine (AUC=0.932) have high diagnostic ability in distinguishing between DN and T2DM. Two oligopeptides, Asn-Met-Cys-Ser and Asn-Cys-Pro-Pro, were correlated with the severity of proteinuria, with AUC values of 0.8857 and 0.9963, respectively, making them potential biomarkers for differentiating the severity of DN (Peng et al., 2022). Through UHPLC-QTOF-ESI-MS analysis of serum and urine from 90 DN patients, arginine (AUC=0.500), L-acetylcarnitine (AUC=0.600), hippuric acid (AUC=0.700), indoxyl sulfate (AUC=0.600), butenoyl carnitine (AUC=0.600), and sorbitol (AUC=0.500) in serum, and p-cresylsulfate (AUC=0.800) in urine may serve as biomarkers for early DN (Balint et al., 2023). In the rat diabetic model constructed by Kikuchi et al. (2019), high levels of phenyl PS were correlated with the severity of glomerular lesions, and a significant correlation between PS levels and ACR was subsequently demonstrated in human plasma. Receiver operating characteristic curve analysis showed that the combined use of PS with known factors increased the AUC from 0.713 to 0.751. These results indicate that the detection of metabolites is helpful for the early diagnosis of DN and assessment of disease severity, and can be used as a disease marker of DN and a target for future treatment.

### 3.3 Metabolism associated with occurrence and development of DN

**Figure 2:**
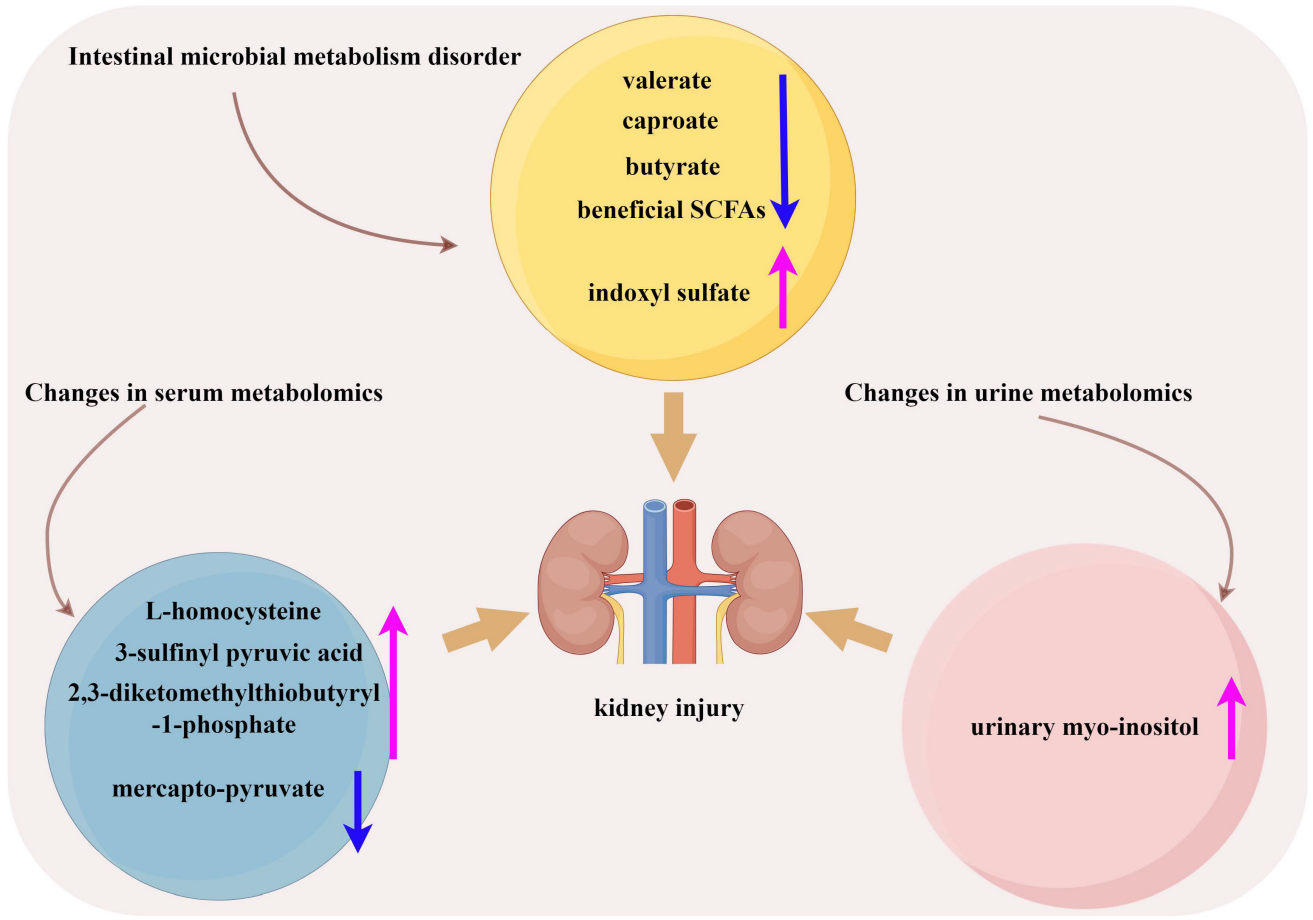
Metabolism associated with development of DN. (By Figdraw).

Disturbance of the gut microbiota in DN patients can disrupt intestinal epithelial function, reduce beneficial SCFA production, and release gut-derived toxins (indoxyl sulfate) and inflammatory factors that can damage the kidneys (Meijers and Evenepoel, 2011). Zhong et al. (2022) confirmed that the decreased levels of gut microbiota metabolites valerate and caproate in DN patients are independently related to the progression of DN and can predict the progression of DN to ESRD (Zhong et al., 2022). Urinary metabolomics analysis revealed an increase in urinary myo-inositol concentration with progression of DN. It showed an additive effect in predicting the progression of ESRD in terms of serum creatinine and urinary protein-to-creatinine ratio (Kwon et al., 2023). In the pathways of cysteine and methionine metabolism, serum L-homocysteine and 3-sulfinyl pyruvic acid, as well as 2,3-diketomethylthiobutyryl-1-phosphate, were elevated in the DN group and increased with the progression of DN proteinuria, while mercapto-pyruvate was decreased in the DN group and further decreased in the heavy proteinuria group (Peng et al., 2022). The level of butyrate was decreased in DN patients, and supplementation with sodium butyrate increased autophagy by activating the AMPK/mTOR pathway in DN rats and improving kidney injury (Cai et al., 2022) (**Figure 2**). Tang et al. (2022) also found a decrease in butyrate levels in DN patients. In db/db mice, supplementation with butyrate can improve intestinal barrier function, activate the PI3K/Akt/mTOR pathway, suppress oxidative stress, and improve muscle atrophy caused by DN. However, some SCFAs have damaging effects on the kidneys. Lu et al. (2020) demonstrated that acetate derived from the gut microbiota activated G-protein-coupled receptor 43, which inhibits AMPKα activity, leading to dysregulation of cholesterol homeostasis and insulin signaling, and progression of DN. Hu Z. et al. (2020) also reached similar conclusions. These results indicate that the metabolites produced by DN patients in different metabolic pathways and different sample types will have different changes, and the role of various types of SCFAs in DN is still controversial. Therefore, more clinical and animal trials are needed to confirm the mechanism of metabolites in DN.

## 4 Gut microbiota and microbial metabolites as therapeutic strategies in treatment of DN

### 4.1 FMT

FMT is the transfer of gut microbiota from healthy individuals to patients with gut microbiota disorders, achieving the goal of rebuilding the homeostasis and diversity of the gut microbiota (Bian et al., 2022). In recent years, FMT has shown specific therapeutic effects in diseases such as migraine (Kappéter et al., 2023), CKD (Liu et al., 2022), and *Clostridium difficile* infection (Wei et al., 2022). After FMT, DN mice showed significant relief of glomerulosclerosis and fibrosis, glomerular injury, basement membrane thickening, and mesangial proliferation, indicating that reconstruction of normal gut microbiota can alleviate DN. In addition, the levels of microbial-derived uremic solutes such as hippuric acid and cholic acid significantly decreased after FMT, indicating that FMT can affect the metabolism of DN mice by regulating microorganisms (Shang et al., 2022). FMT can reduce the destruction of cholesterol homeostasis, thereby improving the damage of renal tubulointerstitium in diabetic rats, suggesting that FMT may be a new strategy for the prevention and treatment of DN (Hu Z. et al., 2020). Another study showed that FMT improved the glomerular injury of streptozotocin (STZ)-induced diabetes in rats (Lu et al., 2021). In a T2DM mouse model, FMT reduced blood sugar, improved glucose tolerance and insulin resistance, and alleviated pancreatic island damage (Wang et al., 2020). These results indicate that FMT may be a new strategy for preventing and treating DN. Although FMT has some potential in the treatment of DN, it is mostly used in animal research, and more clinical trials are needed to confirm its therapeutic efficacy in DN patients, as well as the potential risks.

### 4.2 Diet

A high-fiber diet contributes to the reconstruction of intestinal microorganisms. After the induction of diabetes by a high-fiber diet and STZ, mice had reduced intestinal Firmicutes, increased Bacteroides, and increased Prevotella and Bifidobacterium, which produce SCFAs. This led to increase in concentration of SCFAs in serum and feces, preventing DN through the key pathways and genes involved in innate immunity, inflammation, and macrophage recruitment (Li et al., 2020). It also caused the generation of probiotics and a significant increase in *Akkermansia muciniphila*. A low carbohydrate diet can cause an increase in the abundance of SCFA-producing bacteria (*Roseburis*) and *Ruminococcus* (Liu K. et al., 2023). Intermittent fasting can improve metabolic diseases such as diabetes and cardiovascular disease by improving the composition of gut microbiota (Liu et al., 2020). Dietary polyphenols can stimulate the secretion of glucagon like peptide-1 (GLP-1) by intestinal L cells to improve glucose homeostasis (Wang et al., 2021). Dietary fiber can promote the production of SCFAs by intestinal bacteria, thereby enhancing insulin sensitivity and GLP-1 secretion (Mazhar et al., 2023). These results indicate that adjusting diet can prevent or delay DN by improving gut microbiota and related metabolites, which is worth further exploration.

### 4.3 Probiotics and postbiotics

Probiotics can promote human health by improving intestinal inflammation, regulating gut microbiota homeostasis, repairing cell damage, and regulating immunity, which is important in treating and preventing diseases (Staniszewski and Kordowska-Wiater, 2021; Wolfe et al., 2023). A randomized, double-blind, placebo-controlled trial showed that the intake of probiotics can reduce symptomatic factors by producing SCFAs in the intestine and reducing the production of hydrogen peroxide free radicals, thereby reducing kidney inflammation and fibrosis (Ross, 2022). *Lactobacillus reuteri* GMNL‐263 can reduce hemoglobin A1c and blood glucose levels in rats with STZ-induced diabetes, and inhibit renal fibrosis caused by hyperglycemia (Lu et al., 2010). In a randomized controlled clinical trial, DN patients who consumed soy milk containing *Lactobacillus plantarum* A7 for 8 weeks showed significantly lower levels of cystatin C and inflammatory adipokine progranulin than in the soy milk group (Miraghajani et al., 2019). Supplementing probiotic *Lactobacillus casei* Zhang can improve SCFAs and nicotinamide metabolism, reduce renal injury, and delay renal function decline (Zhu et al., 2021). New compound probiotics (*L. plantarum* and *Lactobacillus delbrueckii* subsp. *bulgaricus*) can serve as adjuncts for metformin by increasing the production of butyrate, enhancing glucose metabolism in patients (Liang et al., 2023). In a mouse model of chronic renal failure induced by hyperglycemia, supplementing probiotics (including TYCA06, BLI-02, and VDD088) can alleviate deterioration of renal function in mice (Kuo et al., 2023). These studies suggest that probiotic supplementation is a potential therapy to improve kidney disease caused by diabetes-related metabolism.

Postbiotics come from metabolites or fragments of microorganisms (such as vitamins, lipids, secondary bile acids, bacteriocins, enzymes, extracellular polysaccharides, and SCFAs), and can also regulate gut microbiota without living microorganisms, resulting in lower intake risk (Gao J. et al., 2019; Żółkiewicz et al., 2020). *Bifidobacterium longum* 35624 can produce an extracellular polysaccharide, which prevents bacterial inflammation and promotes barrier function (Schiavi et al., 2016). When there is a sufficient amount of SCFAs in postbiotic formulations, it can improve epithelial barrier function and protect the body from damage induced by lipopolysaccharides (Feng et al., 2018). In a T2DM rat model treated with postbiotics, heat-inactivated *Streptococcus thermophilus* reduced fasting blood glucose levels, glucose tolerance, and insulin resistance, and increased the abundance of beneficial bacteria such as Ruminococcaceae and *Veillonella* (Gao X. et al., 2019). In a randomized double-blind parallel clinical trial, compared with the placebo group, oral pasteurization of *Lactobacillus griffii* CP2305 significantly increased the content of bifidobacteria in the intestines of the experimental group (Sugawara et al., 2016). The mechanism of action of postbiotics in intestinal diseases has not been fully elucidated, and more clinical trials are needed to verify their effectiveness.

### 4.4 Prebiotics and synbiotics

Prebiotics can regulate glucose metabolism by changing intestinal flora, thus slowing the progress of diabetic complications (Bock et al., 2021). Fructooligosaccharide (FOS) is a common prebiotic. FOS supplementation can improve the renal-related pathological changes caused by diabetes (Pengrattanachot et al., 2022). Similarly, FOS has a protective effect on the kidneys of rats with STZ-induced type 1 diabetes mellitus (T1DM) and improves diabetes-related metabolic abnormalities (Gobinath et al., 2010). Inulin type fructan regulates the gut microbiota of db/db mice, inducing bacterial enrichment that produces SCFAs, leading to an increase in acetate concentration that can improve glomerular injury and renal fibrosis (Luo et al., 2022). Prebiotic supplements can significantly reduce the concentration of uremic toxin cresol sulfate in patients with CKD (Chen L. et al., 2022), increase the level of SCFAs, improve intestinal permeability, and alleviate inflammation (Snelson et al., 2021). Resistant starch (RS) is a prebiotic that promotes the proliferation of beneficial bacteria, such as bifidobacteria and lactobacilli, leading to an increase in SCFA production and a decrease in uremic solutes produced by the microbial community (Snelson et al., 2019). In addition, RS can also alleviate polyuria symptoms and disruption of vitamin D homeostasis in rats with STZ-treated T1DM (Koh et al., 2014).

**Figure 3:**
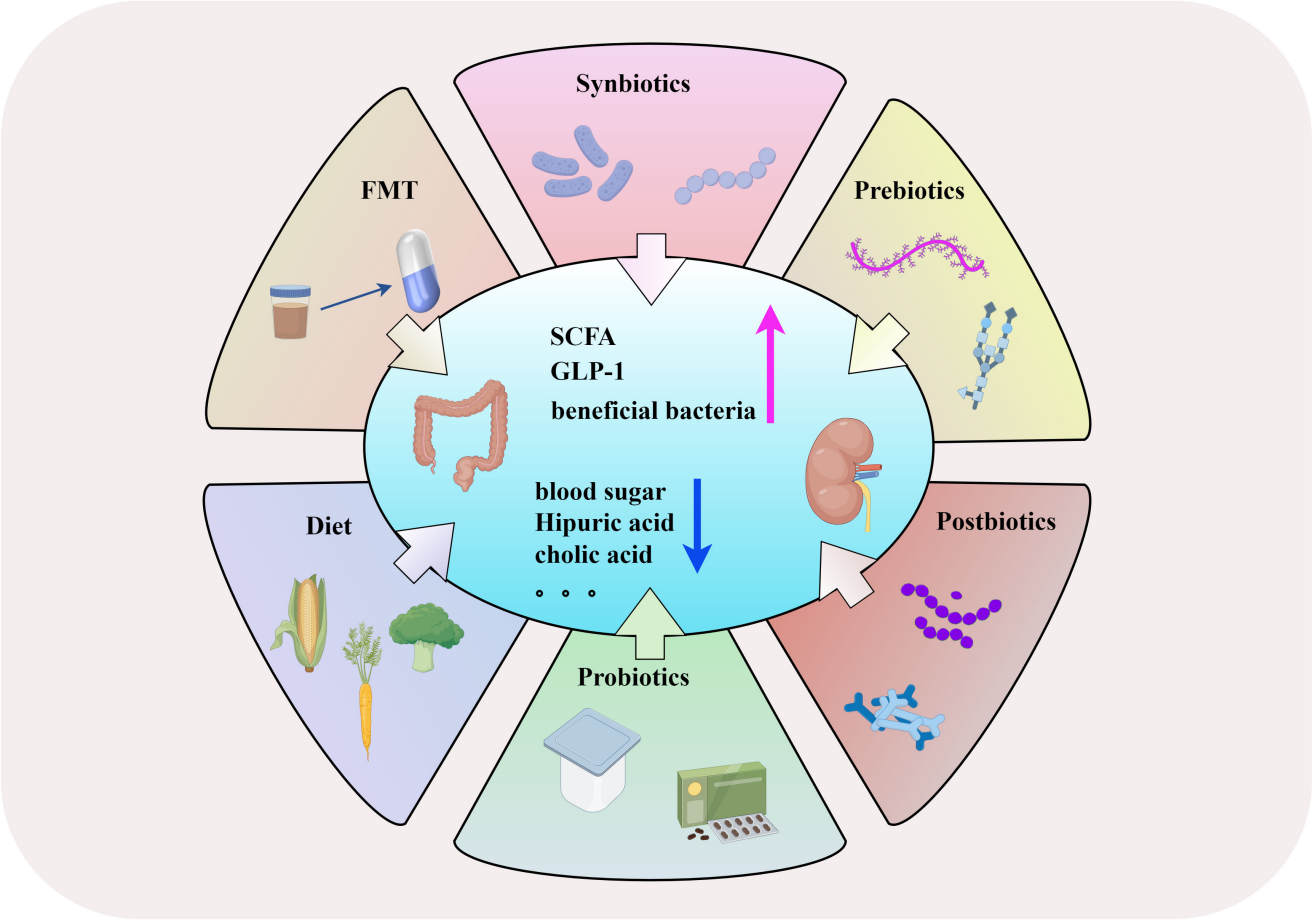
The management and therapeutic strategies of DN based on gut microbiota. (By Figdraw).

Synbiotics are a combination of prebiotics and probiotics. Supplementing synbiotics can improve the composition of intestinal microorganisms and delay the progression of diabetic complications (Jiang et al., 2022). Oral administration of synbiotics (containing *Bifidobacterium lactis* HN019, *Lactobacillus rhamnosus HN001*, and oligofructose) can increase the abundance of beneficial bacteria in the intestine, such as *Clostridium sensu stricto 1*, *Bifidobacterium*, *Lactobacillus*, and *Collinsella* (Li et al., 2023), as well as inhibitory effects on pathogens, increased production of SCFAs, and optimized colon function (Rinninella et al., 2019). In a T2DM model, an increase in SCFA-producing bacteria was observed in rats treated with synbiotics (Mangiferin and *L. reuteri* 1-12) (Meng et al., 2023). However, Liu F. et al. (2023) found that synbiotics cannot reduce serum creatinine levels in nondialysis patients, which may be related to different research subjects and pathogenic factors of kidney disease. At present, there is limited research on synbiotics in DN, and a large number of clinical studies are still needed to confirm their effects (**Figure 3**).

## 5 Conclusion and prospects

In conclusion, we have summarized the composition of gut microbiota and serum and urine metabolites in patients with DN, elucidating the application of microbiota and microbial metabolites in diagnosing DN and their role in disease progression. Kidney damage in DN patients can lead to dysbiosis of the gut microbiota, and disruption of the microbiota can further impair kidney function by producing numerous metabolites, even causing irreversible lesions. Improving the stability of gut microbiota, enhancing glucose metabolism, and reducing the production of uremic toxins by adjusting the structure of the diet, FMT, and oral intake of probiotics/prebiotics can delay the progression of DN.

Despite numerous studies, our understanding of the relationship between DN and gut microbiota and metabolism is still in its early stages. Gut microbiota and microbial metabolites show different patterns in different stages of DN, and the underlying mechanisms are poorly understood. Currently, large-scale clinical studies are not conducted in multiple centers, both domestically and internationally. Evaluating gut microbiota and microbial metabolites as therapeutic strategies in the treatment of DN still requires extensive clinical research for validation. Future research should clarify the specific targets of the impact of gut microbiota and related metabolites on DN, providing new insights for diagnosing and treating DN.

## Author contributions

J-XY: Writing – original draft. XinC: Writing – original draft. S-GZ: Writing – original draft. XiC: Writing – original draft. Y-YW: Writing – original draft. L-PW: Writing – review & editing. S-HX: Writing – review & editing.
